# CRISPR/LbCas12a‐mediated targeted mutation of *Gracilariopsis lemaneiformis* (Rhodophyta)

**DOI:** 10.1111/pbi.13949

**Published:** 2022-11-01

**Authors:** Jingyu Zhang, Qiong Wu, Morgane Eléouët, Rui Chen, Haihong Chen, Ni Zhang, Yiyi Hu, Zhenghong Sui

**Affiliations:** ^1^ Key Laboratory of Marine Genetics and Breeding (Ocean University of China), Ministry of Education Qingdao China; ^2^ Synbio Technologies LLC Suzhou China

**Keywords:** CRISPR/LbCas12a, *Gracilariopsis lemaneiformis*, gene editing, carbonic anhydrase, phycoerythrin, macroalgae

The CRISPR/Cas genome editing system has achieved high popularity in recent years (Knott and Doudna, 2018). It has already been used successfully in several plant species to perform gene knockout (Li *et al*., 2020), activation or repression (Li *et al*., 2019), and to target several sites simultaneously across the genome (Hu *et al*., 2019). In the present study, the CRISPR/LbCas12a (*Lachnospiraceae bacterium* ND2006) system was preliminarily established in *Gracilariopsis lemaneiformis*, an economically important red algae. Several base substitutions, as well as base insertions and deletions upstream of the target site, were detected. The study provides an important reference for the construction of macroalgae gene‐editing systems.

Six targets were initially selected on the carbonic anhydrase sequence (Figure S1a). DNA template for *in vitro* transcript was designed for each target (Figure S1b), synthesized by fill‐in PCR using T7 primer and a unique guide RNA (gRNA) primer (Tables S1 and S2). Subsequently, each gRNA was transcribed *in vitro* (Figure S1c). The activity of LbCas12a was tested on the PCR amplified *ca* fragment (864 bp) combined with pre‐incubated LbCas12a and gRNAs at 25 °C for 30 min (Table S1 CAF&R, Table S2). CAgRNA2 and CAgRNA3 displayed obvious cleavage bands (Figure 1a).

**Figure 1:**
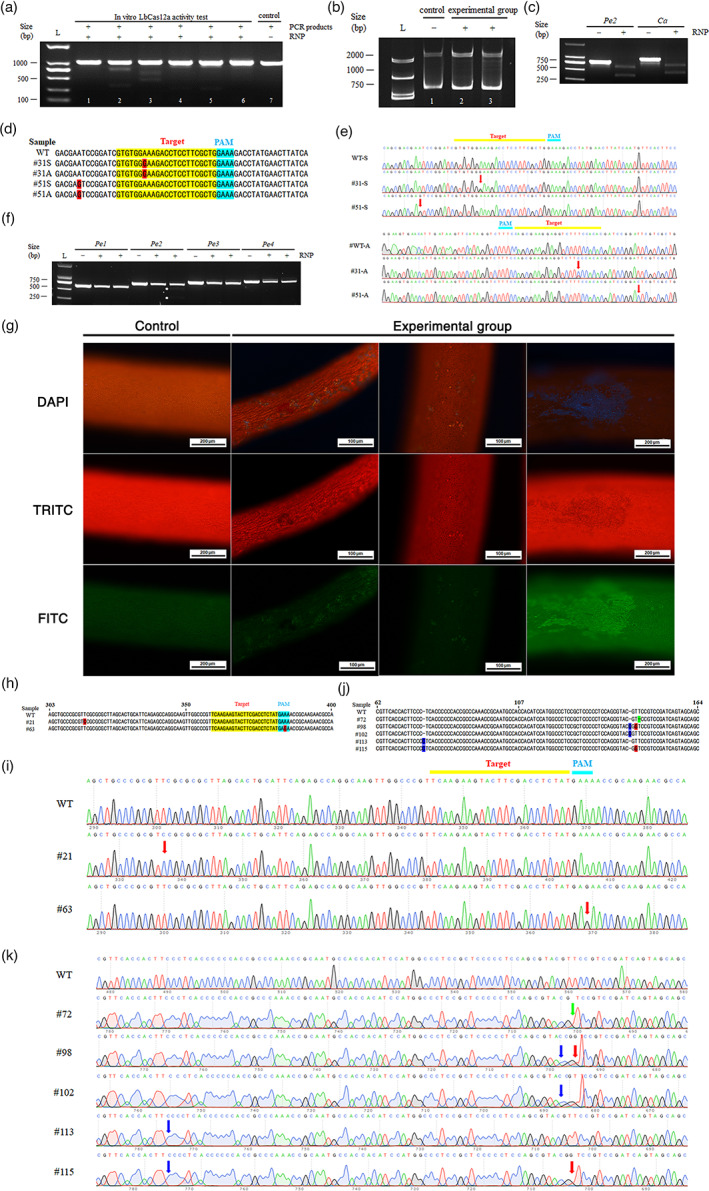
Genome editing of *Gp. lemaneiformis* using the CRISPR/LbCas12a system. (a) *Ca* PCR products (864 bp) were treated with pre‐incubated LbCas12a and gRNAs 1 to 6 respectively (from lane 1 to lane 6). Control was the ca sequence incubated with neither LbCas12a nor gRNA. (b) The electrophoresis results of SSCP (lane 2: CAgRNA2, lane 3: CAgRNA3). The control was wild‐type. (c) The results of *in vitro* editing of CagRNA3 and Pe2gRNA2 combined with Cas12a at 37 °C. (d) The mutation sequence detected by clonal sequencing, in which the reverse sequence underwent reverse complementation. Yellow is the target of gRNA, blue is the PAM sequence and red is the mutation position. (e) Sequencing peak map of wild‐type and mutant sequence; red arrow indicates mutation sites. (f) The results of *in vitro* editing of PCR products for the formation of RNP by Cas12a and gRNA of four phycoerythrin genes at 25 °C. (g) Fluorescence observation of selected spots. Control was a negative group that was only bombarded with gold particles. The experimental group included three groups (vertical rows) of algae tips under different filters after transformation. (h) The mutation sequence detected by clonal sequencing at the position of the target point. Yellow is the target of gRNA, blue is the PAM sequence and red indicates base substitution. (i) *Pe* sequencing peak map of mutant sequence at target point; red arrow indicates base substitution. (j) The mutation sequence detected by clone sequencing at the vicinity of 200 bases upstream of the PAM site; red indicates base substitution, blue indicates base insertion and green indicates base deletion. (k) *Pe* sequencing peak map of mutant sequence at the vicinity of 200 bases upstream of the PAM site, red arrow indicates base substitution, blue arrow indicates base insertion and green arrow indicates base deletion.

Two‐hundred tips of algae were bombardment‐treated by ribonucleoprotein (RNP) complex containing CAgRNA2 and CAgRNA3 (Table S2). Five days later, *ca* gene of the tips was amplified using detection primers (Table S1 CAF&R, Table S2). The mutation sequences were enriched in the PCR products using the Single Strand Conformation Polymorphism (SSCP) technique (Figure 1b). Compared to the control group, weaker different bands were observed in the experimental group. The differential bands were recovered and then subjected to clonal sequencing. Compared to the wild‐type sequence, two sequencing samples with base substitution were observed near the editing site (Figure 1d), one at 17 bases downstream of the Protospacer Adjacent Motif (PAM) site, and the other at 32 bases downstream of the PAM site, both of which were replaced by G from the original A (Figure 1e).

To provide an easily observable trait, a pigment protein, the γ subunits of phycoerythrin (γ*pe*) were also selected as a target gene. In the *Gp. lemaneiformis* (SRR20338037) genome, four sequences annotated as *γpe* were observed (Figures S3–S6). The motif compositions of the protein sequences were very similar; however, the similarity between DNA sequences was low (Figure S1d). For each sequence of *γpe*, two sites were selected, respectively (Figures S3–S6), and the possibility of off‐target effects was reduced through genome alignment. Similarly, the DNA templates for pre‐CRISPR RNA (pre‐crRNA) were generated by fill‐in PCR and transcribed to obtain different gRNAs (Tables S1 and S2). LbCas12a activity was also tested *in vitro* at 25 °C for 30 min (Table S1 PE1F&R, PE2F&R, PE3F&R, PE4F&R, Table S2). Cleavage could be observed in the second gRNA of *pe2* (PE2gRNA2). No obvious cleavage was observed in other experimental groups (Figure 1f). PE2gRNA2 was selected for further activity tests. The LbCas12a activity was then tested at 37°C to ensure that gRNA was effective (Figure 1c). Cleavages could be clearly observed for PE2gRNA2 at 37°C and the products were of the expected sizes (364 and 470 bp).

After microparticle bombardment, the algae were cultured at 25 °C for 2 day, and then cultured at 20 °C. Fifteen days later, the algal tips were checked under fluorescence microscopy. Different spots were observed on the surfaces of the branches. In some areas, the spots were scattered and dotted, while in other areas, patches were formed (Figure 1g). Under different filters, the patches were distinct from the surroundings, with blue, dark, and bright green patches observed under DAPI, TRITC and FITC filters respectively.

The patches were collected using scalpels. *Pe* was amplified by PCR (Table S1 PE2F&R). The sequencing results (Figure 1h,i) showed that near the editing site, base substitution existed in two clones, namely, in the #21 clone, the 67th base upstream of the PAM site gene was shifted from T to C. In the #63 clone, the base substitution occurred at the third base of the PAM site, which shifted from A to G.

In addition, other sequence mutations occurred in the vicinity of 200 bases upstream of the PAM site (Figure S1e). Among them (Figure 1j,k), the 145th base T deletion occurred in the #72 clone. In the #98 clone, base C was inserted after the 142nd base, and the 144th base shifted to G from T. In the #102 clone, base C was inserted after the 142nd base. In the #113 clone, base C was inserted after the 76th base. In the #115 clone, base C was inserted after the 76th base, and 144th base shifted to G from T. The insertion or deletion of single bases in the mutant sequences above led to shifts in the open reading frame, which completely altered the amino acid sequences.

This study, for the first time, established a gene‐editing system for *Gp. lemaneiformis*. Using microparticle bombardment to directly transform RNP into algal tips greatly simplifies the experimental procedures when compared with conventional plasmid systems, and the SSCP method was confirmed to facilitate the screening of editing results in numerous wild‐type cells. To identify gRNAs with high efficiency at 25 °C, more than three gRNA options are required. For macroalgae, this study confirmed for the first time that gene editing could be achieved by the CRISPR/LbCas12a system, and the selection of genes with suitable phenotypes could facilitate the screening of editing results. Our study lays a foundation for gene editing work in *Gp. lemaneiformis* and other macroalgae, and offers key insights.

## Conflict of interest

The authors declare no conflict of interest.

## Author contributions

ZHS, YYH and ME designed the experiments. JYZ, QW, RC, NZ and HHC performed the experiments. ME provided technical supports. JYZ, QW and ME wrote the manuscript. ZHS, YYH and ME supervised the research. All the authors read and approved the manuscript.

## Supporting information


**Appendix S1** Materials and methods.
**Table S1** List of primers.
**Table S2** Reaction systems.
**Figure S1**–**S6** Target gene sequence and target position.
