# Supplementary material for: CRISPR/LbCas12a‐mediated targeted mutation of Gracilariopsis lemaneiformis (Rhodophyta)
Source: Plant Biotechnol J. 2022 Nov 1;21(2):235–7. doi: 10.1111/pbi.13949 (PMC9884009; doi:10.1111/pbi.13949)
Supplement: Supplementary file 1 — Appendix S1 Materials and methods. Table S1 List of primers. Table S2 Reaction systems. Figure S1–S6 Target gene sequence and target position. [file PBI-21-235-s001.docx]

**Supplemental Data**

# CRISPR/LbCas12a-mediated targeted mutation of *Gracilariopsis lemaneiformis* (Rhodophyta)

Jingyu Zhang^1, #^, Qiong Wu^1, #^, Morgane Eléouët^1,2, #^, Rui Chen^1^, Haihong Chen^1^, Ni Zhang^1^, Yiyi Hu^1, *^, Zhenghong Sui^1, *^

^1^ Key Laboratory of Marine Genetics and Breeding (Ocean University of China), Ministry of Education, Qingdao 266003, China

^2^ Synbio Technologies LLC, Suzhou 215000, China

^#^These authors contributed equally to this work.

^*^ Correspondence:

Zhenghong Sui (suizhengh@ouc.edu.cn)

Yiyi Hu (huyiyi@ouc.edu.cn)

**Developing a CRISPR/Cas12a gene editing protocol**

The CRISPR/Cas genome editing system has gained great popularity in recent years thanks to its specificity, simplicity and low cost (Jinek *et al*., 2012). It has already been successfully used in several plant species to perform targeted gene knockout (Lawrenson *et al*., 2015), gene activation or repression (Piatek *et al*., 2015), and to target several sites at a time across the genome (Li *et al*., 2013). The CRISPR/Cas9 technique for gene editing has been applied to a few microalgae species so far (Kwangryul *et al*., 2016; Hopes *et al*., 2016; Nymark *et al*., 2016; Shin *et al*., 2016; wang *et al*., 2016), however, never achieved in macroalgae including red algae. The CRISPR/Cas12a system has advantages over CRISPR/Cas9 including a shorter gRNA required and fewer off-targets events, and the Cas12a nuclease could be used both for gene editing and gene integration.

In addition to the plasmid system, the direct transformation of the protein nucleic acid complex composed of Cas12a and gRNA RNP complexes has been proved to have high transformation efficiency and mutation rate (Kim *et al*., 2016; Woo *et al*., 2015). Firstly, this technique would prevent the risk of plasmid insertion into the genome, and therefore alleviate the risk of off-target effects or mosaicism caused by a prolonged expression of the Cas gene and gRNA, as the RNPs are rapidly degraded after transfection (Woo *et al*., 2015; Kim *et al*., 2014). Secondly, it was also reported that the Cas proteins protected guide RNAs from degradation, while free RNA was quickly degraded (Moreno *et al*., 2017). The advantage of RNPs over plasmids is thus that the gRNA is protected from degradation by the Cas protein, allowing a high mutagenesis efficiency, but that the RNPs is still degraded quickly enough to avoid off-target effects. Finally, there would be no problem of promoter efficiency and there would be no need for optimizing the Cas12a codon usage for *Gp. lemaneiformis*, as the protein would already be purified. Mutagenesis via transfection of Cas12a and gRNA RNP complexes has been successfully achieved in *C. reinhardtii* (Ferenczi *et al*., 2017) and zebrafish (Malzahn *et al*., 2019). The drawback is however that antibiotic selection of transformants cannot be done with this technique except if mutating the target gene confers antibiotic resistance. The mutated individuals have thus to be determined by sequencing, or can also be detected visually by mutating a sequence coding for a protein involved in the regulation of pigmentation like phycoerythrin (Huang *et al*., 2017).

Similar to the delivery of plasmids into *Gp. lemaneiformis* nuclei, delivery of RNPs via microparticle bombardment can be tested. Biolistic delivery of pre-assembled Cas9-gRNA RNPs has for instance successfully been achieved in wheat (Zhen *et al*., 2017) and maize (Svitashev *et al*., 2015). In these instances, Cas9 was incubated with the in vitro transcribed gRNAs before being coated on gold particles and transfected in the cells; a similar protocol can be developed for Cas12a. Guide RNAs for Cas12a can easily be constructed by fill-in PCR and transcribed in vitro (Ferenczi *et al*., 2017). The gRNA associated with Cas12a is called CRISPR RNA (crRNA), and in vitro assays showed that Cas12a is able to process a 69 nt-long pre-crRNA into a shorter 45-50 nt-long mature crRNA, as it possesses both RNAse and DNAse activities (Zetsche *et al*., 2015; Fonfara *et al*., 2016). A trans-activating crRNA (tracrRNA) is thus not needed for the maturation of the crRNA, contrary to Cas9 (Safari *et al*., 2019). The mature crRNA will then guide the target DNA to be cut 18 to 23nt downstream of the PAM (Zetsche *et al*., 2015). Higher targeted mutagenesis was observed in rice when using the pre-crRNA sequence than the mature crRNA (Xu *et al*., 2016), so generating pre-crRNA in vitro before associating it with Cas12a to form RNP complexes could be preference.

Finally, another aspect to consider for designing a CRISPR/Cas12a gene editing protocol in *Gp. lemaneiformis* is the reaction temperature. The activity of the Cas proteins is dependent on temperature, and their activity is reduced below 37°C (Moreno *et al*., 2017; Malzahn *et al*., 2019). It could therefore be a problem for obtaining activity in *Gp. lemaneiformis*, as its ideal growth temperature is 20°C. Cas9 and Cas12a from *Lachnospiraceae* *bacterium* ND2006 (LbCas12a) have, however, shown better efficiency than *Acidaminococcus sp* BV3L6 (AsCas12a) below 28°C, and retain a relatively high activity at 22°C (Moreno *et al*., 2017; Malzahn *et al*., 2019). Therefore, LbCas12a can be used to target *Gp. lemaneiformis* genome and increase HDR frequency.

**Selection of target genes**

The extracellular carbonic anhydrase was first selected as the first target gene, which has the activity of catalyzing the conversion of carbon dioxide and carbonate in the extracellular. *Gp. lemaneiformis* has a mechanism of utilizing HCO_3_^-^ in seawater, which is closely related to extracellular carbonic anhydrase activity (Zou *et al*., 2004). On the one hand, extracellular carbonic anhydrase activity is unnecessary in a ventilated environment, so the loss of this gene function is not lethal to *Gp. lemaneiformis*; on the other hand, by detecting extracellular carbonic anhydrase activity and immunohistochemical techniques to confirm each other, the selection of extracellular carbonic anhydrase gene also helps to verify whether gene editing works in the future. In the genome information of *Gp. lemaneiformis* (SRR20338037), all the genes annotated as carbonic anhydrases were selected, and one of the genes (Fig. S2) with subcellular localization outside the cell was predicted and selected by Plant-mPLoc (Chou and Shen, 2008) (http://www.csbio.sjtu.edu.cn/bioinf/plant-multi/).

In order to facilitate the distinction between transformed cells and normal cells, it is necessary to select a gene that lead to easily observable traits. *Gp. lemaneiformis* has strong autofluorescence under mercury lamp irradiation, which is closely related to various pigments. The phycoerythrin of red algae consists of three subunits, α, β, and γ (Zhang *et al*., 2017), and it is a common chromoplast in red algae and has been modified as a common fluorescent marker. When the function of phycoerythrin is affected, the autofluorescence of *Gp. lemaneiformis* will change, and the color spots which are different from normal cells will appear.

**Materials and methods**

**Plant materials and growth conditions**

The tetrasporophytes were collected in Taiping Jiao Bay, Qingdao, China during their reproductive period in March 2021. The algae were brushed with sterilized seawater until they were free from observable epiphytes. They were cultivated in sterile seawater supply with Provasoli (Pro) medium and grown under a 12h light/12h dark cycle and a light intensity of 30 µmol·m^-2^·s^-^1, at 20±1°C. Among these wild materials, the one with better growth was selected for culture to ensure that the experimental materials have the same genetic background (This material was called WT below). The seawater containing Pro medium was renewed every 5 days.

**Generation of guide pre-crRNA**

Potential guide RNAs (gRNAs) for LbCas12a were chosen with the help of the CRISPRscan online tool (https://www.crisprscan.org/?page=sequence) and restricted to those with TTTV PAM (with V = C or G). Carbonic anhydrase gene (*Ca*) and phycoerythrin gene (*Pe*) were successively selected as target genes for experiments (Fig. S2-S6). No off-targets were detected by BLAST search on the *Gp. lemaneiformis* genome (SRR20338037), and the *Ca* and *Pe* target sequence was verified in WT by Sanger sequencing (see primer list). Double strand template for gRNA transcription in vitro was synthesized by fill-in PCR with specific primers and universal primers. The specific primers were designed by putting together (from 5' to 3') a poly A sequence, the 20- nucleotide long spacer (target sequence) identified by CRISPRscan, and the 21-nucleotide long repeat sequence of the LbCas12a pre-crRNA. The poly A sequence was added in order to create an uridinylate-rich 3' overhang in the crRNA, as it was shown to increase editing efficiency. The LbCas12a-pre-crRNA universal primer was designed by putting together (from 5' to 3') the T7 promoter sequence preceded by three cytosines and followed by two guanines, and the 36- nucleotide long LbCas12a pre-crRNA sequence (see primer list). The primers were ordered from Sangon Biotech.

A fill-in PCR (Tab. S2. Fill in PCR) was then carried out using one of the specific primers and the T7 primer (See List of primers). The fill-in PCR was repeated for each specific primer. The product after fill-in PCR is shown in the following. **X** represents six different target sequences:

5'CCCTAATACGACTCACTATAGGGTTTCAAAGATTAAA**TAATTTCTACTAAGTGTAGAT**XXXXXXXXXXXXXXXXXXXXXXXTTTTATTTTTT3'

3'GGGATTATGCTGAGTGATATCCCAAAGTTTCTAATTT**ATTAAAGATGATTCACATCTA**XXXXXXXXXXXXXXXXXXXXXXXAAAATAAAAAA5'

Grey is poly A tail, which can improve the editing efficiency of Cas 12a, green is gRNA target sequence, and blue is pre-crRNA repeat sequence. In universal primers, yellow is T7 promoter.

Each fill-in PCR products was then transcribed using the Invitrogen MAXIscript T7 in vitro transcription kit (Thermo Fisher, USA), with the T7 RNA polymerase, following the manufacturer's instructions (Tab. S2. In vitro transcription). The post-transcriptional product is as follows:

5'GUUUCAAAGAUUAAA**UAAUUUCUACUAAGUGUAGAU**XXXXXXXXXXXXXXXXXXXXXXXUUUUAUUUUUU3'

Turbo DNase was then added to the mix in order to remove the DNA. The obtained RNA was then cleaned by adding 30µl of ddH_2_O and 5µl of 5M Ammonium Acetate. After having added 150 µl of 100% EtOH to each mix, they were chilled at -20°C for 30 min and spinned for 20 min at 12000 rpm at 4°C. The supernatant was discarded and the pellet was washed with 70% chilled EtOH. Each pellet was resuspended in 20 µl nuclease-free ddH_2_O, and 1 µl of 0.5 M EDTA was added to each tube. The RNA was then stored at -80°C.

***In vitro* Cas12a activity test**

1 µM LbCas12a (ordered from NEB, USA) was pre-incubated with each of the gRNAs (pre-crRNAs) for 10 min at room temperature, before being incubated with pre-amplified *Ca* and *Pe* PCR fragment at 25°C or 37℃ for 30 min (Tab.S2. In vitro Cas12a activity test). 1 µl of 20 mg/ml proteinase K was added to the mix, incubated at room temperature for 10 min, and cleavage of the PCR fragment by the RNPs was checked on a 1.5% agarose gel electrophoresis.

**Microparticle bombardment**

30 mg of 0.8-1.0 µm diameter gold particles (Bio-Rad, USA) were suspended in 1 ml of 70% ethanol and vortexed for 5 min. After letting them stand in the solution for 15 min at room temperature, they were centrifuged at 1500 rpm for 5 min. The supernatant was then discarded and the particles were washed 3 times by adding 1 ml of sterile water, vortexed for 1 min, and centrifuged at 1500 rpm for 5 min. The supernatant was then discarded and the particles were resuspended in 500 µl of 50% glycerol. They were stored at -20°C.

5 µl of the gold particle suspension were centrifuged at 10000 rpm for 10 sec and the supernatant was removed. They were washed three times with 1 ml double-distilled water (ddH_2_O) and resuspended in 42.5 µl of ddH_2_O. 100 µM LbCas12a (ordered from NEB, USA) was pre-incubated with each of the gRNAs for 10 min at room temperature. 5 µl of the washed gold particles were added to each of the 28 µl of pre-incubated LbCas12a and gRNA. (Tab. S2. Microparticle bombardment)

Bombardment was performed using a PDS-1000/He biolistic delivery system (Bio-Rad, USA). The coated gold particles were spread on the bombardment carrier and let air-dried for 1h at room temperature. Bombardments were performed according to the manufacturer’s instructions. The 1cm algal tips were placed at a distance of 6 cm from the firing disc, and then the gold microparticles carrying RNPs were bombarded under the conditions of 900 pounds per square inch under vacuum conditions of 28 pounds per square inch. Each of the RNPs was prepared twice, once for incubation at 25°C (for two hours before being moved at 20°C) and once for incubation at 20°C after bombardment. Controls were uncoated gold particles. One hour after bombardment, the tips were grown in seawater complemented with Pro medium. DNA was extracted 5 days after bombardment.

**DNA extraction and PCR-SSCP analyses**

Total DNA was extracted by the Plant Genomic DNA Kit (TIANGEN, China) and PCR was carried out on the *Ca* gene by detection primers (see primer list). PCR was performed in 50 μl reactions using 1 μl (approximately 30–50 ng) of extracted DNA solution as the template. The PCR fragment was purified by the EasyPure Quick Gel Extraction Kit (Trans, China). The sample of 10 μl fragment and 10 μl 2X SSCP Deionized Formamide Gel Loading Buffer (Sangon Biotech, China) was heated at 100℃ For 10 min, and then was loaded onto non-denaturing 6% polyacrylamide gel and subjected to electrophoresis in 1×TBE buffer. The fragment was collected by the E.Z.N.A Poly-Gel DNA Extraction Kit (OMEGA BIO-TEK, USA).

**Reference**

Dinghui Zou, Jianrong Xia, Yufeng Yang. Photosynthetic use of exogenous inorganic carbon in the agarophyte *Gracilaria lemaneiformis* (Rhodophyta)[J]. Aquaculture, 2004, 237: 421-431.

Ferenczi A, Pyott D E, Xipnitou A, et al. Efficient targeted DNA editing and replacement in Chlamydomonas reinhardtii using Cpf1 ribonucleoproteins and single-stranded DNA[J]. Proc Natl Acad Sci U S A, 2017, 114(51):13567-13572.

Fonfara I, Richter H, Bratovic M, et al. The CRISPR-associated DNA-cleaving enzyme Cpf1 also processes precursor CRISPR RNA.[J]. Nature, 2016, 532(7600):517.

Hopes A, Nekrasov V, Kamoun S, et al. Editing of the urease gene by CRISPR-Cas in the diatom Thalassiosira pseudonana[J]. Plant Methods, 2016, 12(1):49.

Huang X, Zang X, Wu F, et al. Transcriptome Sequencing of Gracilariopsis lemaneiformis to Analyze the Genes Related to Optically Active Phycoerythrin Synthesis[J]. Plos One, 2017, 12(1): e0170855.

Jinek M, Chylinski K, Fonfara I, et al. A Programmable Dual-RNA–Guided DNA Endonuclease in Adaptive Bacterial Immunity[J]. Science, 2012, 337(6096): 816-821.

Jun Zhang, Jianfei Ma, et al. Structure of phycobilisome from the red alga *Griffithsia pacifica* [J]. Nature, 2017, 551(7678):57-63.

Kim H., Kim H.T., Ryu J., Kang B.C., Kim J.S., Kim S.G. (2016). CRISPR/Cpf1 -mediated DNA-free plant genome editing. Nat. Commun. 16(8):14406.

Kim S., Kim D., Cho S.W., Kim J., Kim, J.S. (2014). Highly efficient RNA-guided genome editing in human cells via delivery of purified Cas9 ribonucleoproteins. Genome Res. 24:1012–1019.

Kuo-Chen Chou and Hong-Bin Shen. Cell-PLoc: A package of web-servers for predicting subcellular localization of proteins in various organisms [J]. Nature Protocols, 2008, 3: 153-162.

Kwangryul, Baek, D Hyoung, et al. DNA-free two-gene knockout in Chlamydomonas reinhardtii via CRISPR-Cas9 ribonucleoproteins. [J]. Scientific reports, 2016.

Lawrenson T, Shorinola O, Stacey N, et al. Additional file 3: of Induction of targeted, heritable mutations in barley and Brassica oleracea using RNA-guided Cas9 nuclease. 2015.

Li J F, Norville J E, Aach J, et al. Multiplex and homologous recombination-mediated genome editing in Arabidopsis and Nicotiana benthamiana using guide RNA and Cas9. [J]. Nature Biotechnology, 2013, 31(8):688-91.

Malzahn A A, Tang X, Lee K, et al. Application of CRISPR-Cas12a temperature sensitivity for improved genome editing in rice, maize, and Arabidopsis[J]. BMC Biology, 2019, 17(1):1-9.

Moreno-Mateos M A, Fernandez J P, Rouet R, et al. CRISPR-Cpf1 mediates efficient homology-directed repair and temperature-controlled genome editing[J]. Nature Communications, 2017, 8(1):2024.

Nymark M, Sharma A K, Sparstad T, et al. A CRISPR/Cas9 system adapted for gene editing in marine algae[J]. Rep, 2016, 6:24951.

Piatek A, Ali Z, Baazim H, et al. RNA-guided transcriptional regulation in planta via synthetic dCas9-based transcription factors. [J]. Plant Biotechnology Journal, 2015, 13(4):578-589.

Safari F, Zare K, Negahdaripour M, et al. CRISPR Cpf1 proteins: Structure, function and implications for genome editing[J]. Cell and Bioscience, 2019, 9(1).

Shin S E, Lim J M, Koh H G, et al. CRISPR/Cas9-induced knockout and knock-in mutations in Chlamydomonas reinhardtii[J]. Rep, 2016, 6:27810.

Svitashev S, Young J, Schwartz C, et al. Targeted Mutagenesis, Precise Gene Editing, and Site-Specific Gene Insertion in Maize Using Cas9 and Guide RNA[J]. Plant Physiology, 2015:931-945.

Wang, Qintao, Lu, et al. Genome editing of model oleaginous microalgae Nannochloropsis spp. by CRISPR/Cas9. [J]. Plant Journal, 2016

Woo J W, Kim J, Kwon S I, et al. DNA-free genome editing in plants with preassembled CRISPR-Cas9 ribonucleoproteins[J]. Nature Biotechnology.

Xu R, Qin R, Hao L, et al. Generation of targeted mutant rice using a CRISPR-Cpf1 system[J]. Plant Biotechnology Journal, 2016, 15(6).Zetsche B, Gootenberg J, Abudayyeh O, et al. Cpf1 Is a Single RNA-Guided Endonuclease of a Class 2 CRISPR-Cas System[J]. Cell, 2015, 163(3).

Zhen L, Chen K, Li T, et al. Efficient DNA-free genome editing of bread wheat using CRISPR/Cas9 ribonucleoprotein complexes[J]. Nature Communications, 2017, 8(1):14261.

**
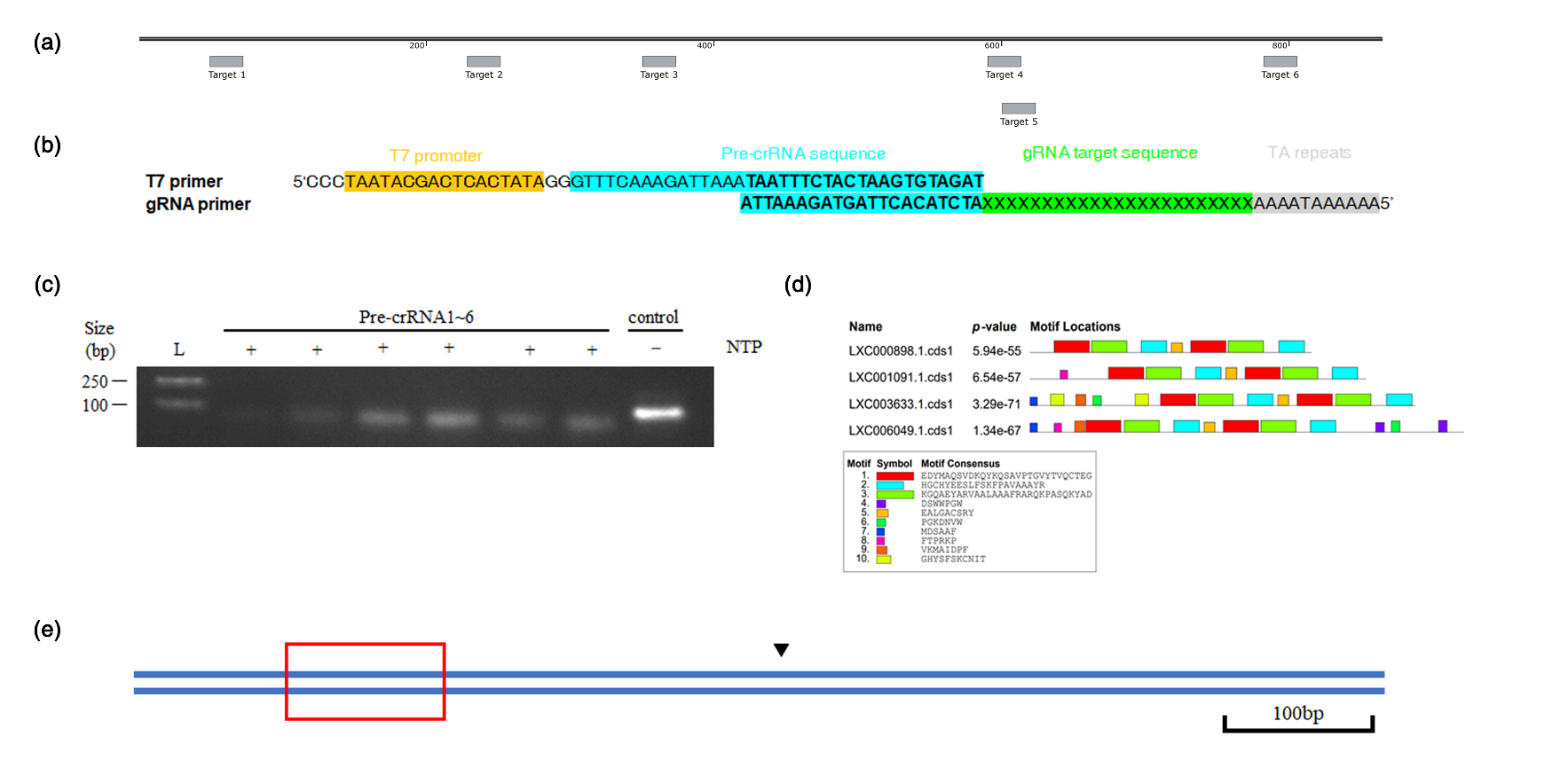
**

**Fig. S1. Additional details on experimental content** (a) There are 6 target sites as candidates in carbonic anhydrase gene. Their starting positions are located at positions 49, 228, 350, 590, 600 and 782 of carbonic anhydrase, respectively. At the same time, these loci were also excluded of nonspecificity through genome blast. (b) The DNA templates for pre-crRNA were generated by fill-in PCR. Every different gRNA primer (such as Tab.S1 CAgRNA1-6) and T7 primer (Tab. S1) form a local double strand through base pairing, and then a double strand containing T7 promoter, Pre-crRNA sequence, gRNA target sequence and TA repeats were amplified under the action of Taq enzyme. (c) The quality of crRNAs produced were checked by gel electrophoresis. Control was the templates incubated with T7 RNA polymerase without NTP. (d) Four kinds of γ subunit of phycoerythrin were found from the genome of *Gp. lemaneiformis* (SRR20338037). The similarity of these DNA sequences was very low, but the motif composition was very similar. (e) DNA double strands of phycoerythrin γ subunit, black triangle indicates the position of target point, red box indicates the position of sequence mutation upstream target point.

ATGAACTTCTTCGCTACCGCCTCCGTCTTTCTACTCTTCTTGTCCGTCGCCCTCCCCGTGCGGGCGAACGAGGAAATCGAGGTCGACACCGCCGGCTGCCCCGTCAAGAGCGCCCAAGGAGACTTCAGTTATGACATGACTCTTCCAAACAACCCCACCCGCTGGGGCGACATCAAGGAGGACTTCGCCACATGCAAGGAAGGAGAAAAGCAATCGCCCATCAACTTCCCCGCCAACGTTCAATACGCCCCGAAATCGGCCGGACCCAAGTCTCAAATGTCGCTCGCAAACATGACCTTTGGCGCCTCCTCCTACAACTGGGCCATGTCCTGCAGCGACGAATCCGGATCGTGTGGAAAGACCTCCTTCGCTGGAAAGACCTATGAACTTATCAATGTTCACTTCCACTCCCCGAGTGAGCACAAGTTGTTCGGCAAGGAATATCCGCTTGAATGTCACATGGTGCATGCTGCCGAGGATGGATCGCTTGCTGTGGTCGGAATGATGTTTGAGTACGCCGAACAAACCTCCTATCCCGCCAGAATTTACCAGAACGTCGTTGAGGAATATGGCGACAACGTCGTCTTTTCCACTATTTTGGGAGGCGTCAAGAGCGATCGTGGAGAATGGGCTGTGCCGGTGGGTCAACTGGTCAACCATAACAAGGGATATTGCTCCTACTCCGGGTCGCTCACCACACCGCCTTGCACTGAAAGTGTCACCTGGTTCATGTCTATGAACATCTGGACCGTGTCTGAACGACAGGTGCATGACTACGTCCGAACGGTTGGCACCAGCATTGAGGGAAATCATCGACCGGTTCAACCACTCAACGAACGTCCGGTCACTTGCTACGTGTCCTAA

**Fig. S2. Carbonic anhydrase α- *CA*-1 complete ORF**. Blue (blue) and brown (brown) are positive chain editing sites and PAM respectively, green and yellow are negative chain editing sites and PAM respectively. In order to facilitate the identification of overlapping parts, CAgRNA5 was labeled with black box.

ATGCCCCCATTCAAAGGGGATCCGGACACTACGATCAACCGCAATTATATGCAGGATGCAGATAGATATATGGCGCAGTGTATAACGATGCAGTACAAGATGACTGCGGCTCCCCTTGGTGTGTACAATGTGCAATGCACCGAAGGAACGATACGCGGCCAAGCGGAAGATGCGCGGAATCTTGCCTTGTCAACCAGCTTCCGTATGAAGCAGCGCACTGTCTCACAGAAATTCGCCGACTACACAGAGACGCGACGCAAAGCTCTTATCGGAGCTCACGGCTGCACATACGAAGAAAAGCTGTTGGCCAAATTCCCTATCTCGGCGCGCGCTTACGTGCGCTCAGGCGCAGAGGCAAAGAGCACCTGTACGCGCTACGCAAATGGGGCTACTGCGGCCGAGAAGTACATGGCGGCATGCGTGGACAAGCAAAGTATGTCACGACTAGTGCCAACCGGAGTGTATGGCGTGACTTGCAACGATGGAAATACAAAGCAAGTTGCTGAGTACAAGAGGGTTCAAGCTCTAGCGGCCAAGTTCAGAGCGAACCAACAACCCTCACTCGTCAAAGAGAGTATCAAGTTTGAGAGTGCAAAGTACGCACGGGACTACTTTGGGCACCTGTGCAGTTACGAGGAAAGCTTGTTCAATTCTTTTCCAGCTGTGGCGGCGTCTATGCGCCCCAGTATCTCGTACTGA

**Fig. S3. Phycoerythrin LXC000898 (*pe*1) complete ORF**. Green and yellow are negative chain editing sites and PAM respectively.

ATGTTCGCTTTCGCCCCATCCGTGTCCGTCACTGCTGCCCGCACCACCTTGCCTTCCACTTCGTTCACCACTTCCCTCACCCCCCACCGCCCAAACCGCAATGCCACCACATCCATGGCCCTCCGCTCCCCCTCCAGCGTACGTTCCGTCCGATCAGTAGCAGCCGCCCGAGCCTCCGACATTCTCGCCAAGGCCGCCGACTACATGGCCAATTCCGTTCTCTACCAGTACTACAACATTGCCAACCCCACCGGCGAGTACGGCGTCCAGTGCACGGAGGGTTCCGTCAAGGGAGCCGCAGAAGCTGCCCGCGTTCGCGCGCTTAGCACTGCATTCAGAGCCAGGCAAGTTGGCCCGTTCAAGAAGTACTTCGACCTCTATGAAAACCGCAAGAACGCCATTGCCGCCGATCATATCTGTCAGTATGAGGAGACGCTCTTCTCGCGCTACCCGAGCGTGGCCGCCACGTACAACGTTGCGCGCAACGAGGCAAATGGTGCTTGCAGTCGCTACGCCACCCCGGAGAGTGTTGAGGAAGCTGCCATGTTGCGTTACATGGACATTCAACAGAACAATGCTGCCAACCCCTCAGGCGTGTACAACTCCTCGTGCAACGAGGGAGCAGCCAAGGGACAATCCGAGCATCTGCGCATCGCCGCCCTCAACGTCGCCTACAGAAACGCTCAAAAGCCCCTCGCCCAGATCTTGCAGGAGAAGTACGAGCAGAAGAAGTACGGATACGTTCAGTGCCATGGCTGCAACTATGAGGAATCGCTTGTTAGTAAGTTCCCCGCTATTGGAGCTGCCTTCCGTGCAAAGACCTATGGCTATTAG

**Fig. S4. Phycoerythrin LXC001091 (*pe*2) complete ORF**. Blue and brown are positive chain editing sites and PAM respectively, green and yellow are negative chain editing sites and PAM respectively.

ATGGACTCCGCCGCTTTTGCCGTAAACGGAATGTTCTCTGCCGTCAACGTCGGAACCTCTTCCTTCACCAAGAACAATGTCACCTCGCAAAGAACCACAGCCTCCCCCGCCGCCGTCCGCATGGCCGTCGATCCTTTCCAGAAGCAATTCCAGTCCCCCGGAAAGATAAACGTTGACTACTCCCGTCCCAAGAAGCTCGCCACCTACAAGCGAAGTGGCTACTCCGCCATCCTCGACTACCCGACCCAACCCTCCATGGCCGGTCACTACTCCATCTCCAACTGCAACATCACCAGCGGCGCCAAGAAGATTCTCATGAAGTACGACGAGTACTGCGCAAAGGGAATGATGCAGGTCTACAAGCGTTCAGCCGTGCCGTACGGAGAGTACACCACCAAGTGCACCGAGGGAACCCTTCCGCAACAAGCTTTTGACAAGCGCGTTTTCAACCGCACCCAGGCATTCCGCCAGGCTCAGAAGCCCATCAATGTTCGCCTTGGTGAGCAGTACGAGAACCGTCGTCTTGCATTCATTTTCACCAACGGCTGCCACCGTGAGGAACAGCAGTTCAAGGAAATGCCCATGTCCACTGCCACCTACCTGGCCGGACGTTCCGAAGCTCTTGGAACCTGCTACAGACTCGTCACTCCAACTACCGTTGCAGAGGATTATATGGCCGACAGTGTGCGCTCCCAGATCACTCAAAAGGCTCACCCGACCGGTGTCTACCGCGTCGGAGTCTGCGAAGATGGTTACGCCAAGGGTGACGCTGAGAACAGACGTGTCGCCGCTCTTGCCTCCGAGTTCCGTACCAGCCAGCAATCCGCCTCTGCTATCACTGGTCAGCAGTACGAATCTGCGCGCACTGCACGCAAGCTCTATGCCAGCTCTTGCCACCACGAGGAAACTCAAATCTACCAGTACCCGGCTGTTGCCGCTGCTATGTGCCGCGATTAA

**Fig. S5. Phycoerythrin LXC003633 (*pe*3) complete ORF**. Green and yellow are negative chain editing sites and PAM respectively.

ATGCATTCCGCCGCGTTCACCACCCCGGTTGTCTTGCAATCCACCGCCAATTCTTCCTCCTTCACGCCGCGCAAACCGCTGTCCGCGCGCAATGCAACAAGCACGCCATTGGTAAAGATGGCCATCACGCCCATTGCAGACATTTTTCAAAACCGTTCTCAAACCCGGGAAGCAAAGCAATCCGCCGCATCCAAAGGCGTGTACACCGTGCAATGCACAGAGGGCACAGCCGGTGCCAACACAGCTGAATACTCGCGTCTATCCTCTCTGGCGCGCTCATTTCGACTTCGTCAAAGTAGCGCTTCCGCCCGATACGCAGACTTGTTCGCCACTCGTCGCGCCGCTGTTATCGCCGCTGCAGGCTCGCATGTCGAAGAAAGTTACGCCGTGCGTTTCCCAGCACGCGCAGCGGCTACGGTTGCCTCCCGCGCTGAGAAACTTCGCGCCTGCTCTCGATACATCGACGCCGCTGATGAGGCCGAACAGTACATGTTTCAGTGCGTGGACAATCAATATAATGCACTCAAGGTACCCGGCGGGGTGTACTCCGTGCAGGCATGCAACGCACGTCAATCTGATGACGTTGACACCGCGCGCGTGTGTGCAGGAGCTGCCGTGTTTCGCTCAAATCAGTTGAGCAATTCACAAAAGACGCAGCAACGTTATAATGCATCTCTGGAGGCCATTTATTCCGGCCGCGGGTGCACCTACGAAGAGGACGAATACATGAACTTCCCGAAGATGGCAGGCGCCATTCGTTGGTCCACAGGTGCTTACGCGGCGTCTGTGTCGAGTGTGCAGGGCGCTATGAATGTGCGTGTCCCAAGCGTAACAGAGCGTATTCAAGGCGTCAACCGTGACAGCTTTTGGCCCAGCTACAAGATACGTGAGGCTGTTCCGAGGAAGGATCCAGTTTGGAAGGCTCCAAGTGTGAAGAACTACGCGCCCATGAGTGCGGCTGCGTTGCAATACGGAATTGATGCGCAGACCAAGCAGCCGGAGTTGCCTTCCTACGACAGCTGGAAGCCCGGCTGGGCGCCCAAGTCTTCCATCAAGTTGAGCCCATATGAACGTTGA

**Fig. S6. Phycoerythrin LXC006049 (*pe*4) complete ORF**. Green and yellow are negative chain editing sites and PAM respectively.

**Tab. S1. List of primers**

| Primer name | Primer sequence (5'-3') | Application |
| --- | --- | --- |
| CAF | CCATCCCATCGCAACTCACA | *Ca* PCR and sequencing  *Ca* PCR and sequencing |
| CAR | GAGGAGGACAAACAATTAGGACAC |  |
| CAgRNA1 | AAAAAATAAAACCCTCCCCGTGCGGGCGAACGAGATCTACACTTAGTAGAAATTA | gRNA primer, used for synthesis of in vitro transcript |
| CAgRNA2 | AAAAAATAAAACCCGCCAACGTTCAATACGCCCCATCTACACTTAGTAGAAATTA | gRNA primer, used for synthesis of in vitro transcript |
| CAgRNA3 | AAAAAATAAAAGTGTGGAAAGACCTCCTTCGCTGATCTACACTTAGTAGAAATTA | gRNA primer, used for synthesis of in vitro transcript |
| CAgRNA4 | AAAAAATAAAATCTTGACGCCTCCCAAAATAGTGATCTACACTTAGTAGAAATTA | gRNA primer, used for synthesis of in vitro transcript |
| CAgRNA5 | AAAAAATAAAACCACGATCGCTCTTGACGCCTCCATCTACACTTAGTAGAAATTA | gRNA primer, used for synthesis of in vitro transcript |
| CAgRNA6 | AAAAAATAAAAAACGGTTGGCACCAGCATTGAGGATCTACACTTAGTAGAAATTA | gRNA primer, used for synthesis of in vitro transcript |
| T7 primer | CCCTAATACGACTCACTATAGGGTTTCAAAGATTAAATAATTTCTACTAAGTGTAGAT | gRNA primer, used for synthesis of in vitro transcript |
| PE1F | GACACTACGATCAACCGCAATT | *Pe1* PCR and sequencing |
| PE1R | GCATAGACGCCGCCACA | *Pe1* PCR and sequencing |
| PE2F | GTTCGCTTTCGCCCCATC | *Pe2* PCR and sequencing |
| PE2R | GCTCCAATAGCGGGGAACTT | *Pe2* PCR and sequencing |
| PE3F | CGCCGCTTTTGCCGTAA | *Pe3* PCR and sequencing |
| PE3R | GGTAGATTTGAGTTTCCTCGTGGT | *Pe3* PCR and sequencing |
| PE4F | CGCAAACCGCTGTCCG | *Pe4* PCR and sequencing |
| PE4R | TCATATGGGCTCAACTTGATGG | *Pe4* PCR and sequencing |
| PE1gRNA1 | AAAAAATAAAAGCCGACTACACAGAGACGCGACGATCTACACTTAGTAGAAATTA | gRNA primer, used for synthesis of in vitro transcript |
| PE1gRNA2 | AAAAAATAAAAGAGCTCACGGCTGCACATACGAAATCTACACTTAGTAGAAATTA | gRNA primer, used for synthesis of in vitro transcript |
| PE2gRNA1 | AAAAAATAAAAGCAGTGACGGACACGGATGGGGCATCTACACTTAGTAGAAATTA | gRNA primer, used for synthesis of in vitro transcript |
| PE2gRNA2 | AAAAAATAAAATCAAGAAGTACTTCGACCTCTATATCTACACTTAGTAGAAATTA | gRNA primer, used for synthesis of in vitro transcript |
| PE3gRNA1 | AAAAAATAAAATCACCAAGAACAATGTCACCTCGATCTACACTTAGTAGAAATTA | gRNA primer, used for synthesis of in vitro transcript |
| PE3gRNA2 | AAAAAATAAAACCAGAAGCAATTCCAGTCCCCCGATCTACACTTAGTAGAAATTA | gRNA primer, used for synthesis of in vitro transcript |
| PE4gRNA1 | AAAAAATAAAACAATGCAACAAGCACGCCATTGGATCTACACTTAGTAGAAATTA | gRNA primer, used for synthesis of in vitro transcript |
| PE4gRNA2 | AAAAAATAAAAAAACCGTTCTCAAACCCGGGAAGATCTACACTTAGTAGAAATTA | gRNA primer, used for synthesis of in vitro transcript |

**Tab. S2. Reaction systems**

| **reaction components** | | | **reaction condition** | | | |
| --- | --- | --- | --- | --- | --- | --- |
| **Application** | **Components** | **Dosage** | **Steps** | **Temperature** | **Time** | **Number of cycles** |
| *Ca* PCR and sequencing | Primer CAF | 2 μl | Initial denaturation | 95℃ | 5 min | 34 |
|  | Primer CAR | 2 μl | Denaturation | 95℃ | 15s |  |
|  | Phanta Max Master Mix (Vazyme, China) | 25 μl | Annealing | 60℃ | 15s |  |
|  | ddH_2_O | 19 μl | Extension | 72℃ | 30s |  |
|  | WT DNA | 2 μl | Final extension | 72℃ | 5 min |  |
| *Pe* PCR and sequencing | Primer PEF | 2 μl | Initial denaturation | 95℃ | 5 min | 34 |
|  | Primer PER | 2 μl | Denaturation | 95℃ | 15s |  |
|  | Phanta Max Master Mix (Vazyme, China) | 25 μl | Annealing | 60℃ | 15s |  |
|  | ddH_2_O | 19 μl | Extension | 72℃ | 30s |  |
|  | WT DNA | 2 μl | Final extension | 72℃ | 5 min |  |
| Fill in PCR | gRNA primer | 2 μl | Denaturation | 95℃ | 15s | 27 |
|  | T7 primer | 2 μl | Annealing | 60℃ | 15s |  |
|  | Phanta Max Master Mix (Vazyme, China) | 25 μl | Extension | 72℃ | 30s |  |
|  | ddH_2_O | 21 μl | Final extension | 72℃ | 5 min |  |
| In vitro transcription | ddH_2_O | up to 20 μl | Transcription | 37℃ | 1 hour | - |
|  | DNA template | 1 μg | Add 1 μl TURBO DNase I | 37℃ | 15 min |  |
|  | Buffer | 2 μl |  |  |  |  |
|  | ATP, CTP, GTP, UTP | 1 μl each | Add 1 μl 0.5M EDTA | 65℃ | 10min |  |
|  | T7 enzyme | 2 μl |  |  |  |  |
| In vitro Cas12a activity test | gRNA | 30 ng | pre-incubated | room temperature | 10 min | - |
|  | 1μM LbCas12a (NEB, USA) | 1 μl |  |  |  |  |
|  | NEB 2.1 buffer | 3 μl |  |  |  |  |
|  | ddH_2_O | Up to 30μl | Incubation | 25℃ | 30 min | - |
|  | PCR fragment | 2500 ng |  |  |  |  |
| Microparticle bombardment | gRNA | 4 μg | pre-incubated | room temperature | 10 min | - |
|  | 100μM LbCas12a (NEB, USA) | 2 μl |  |  |  |  |
|  | NEB 2.1 buffer | 3 μl |  |  |  |  |
|  | ddH_2_O | Up to 35μl |  |  |  |  |
|  | gold particles | 5 ul |  |  |  |  |
